# Microcantilever Actuation by Laser Induced Photoacoustic Waves

**DOI:** 10.1038/srep19935

**Published:** 2016-01-27

**Authors:** Naikun Gao, Dongfang Zhao, Ran Jia, Duo Liu

**Affiliations:** 1 State Key Laboratory of Crystal Materials, Shandong University, 27 South Shanda Road, Jinan, Shandong 250100, P. R. China

## Abstract

We present here a combined theoretical and experimental investigation on effective excitation of microcantilever by using photoacoustic waves. The photoacoustic waves arose from a vibrating Al foil induced by an intensity-modulated laser. We demonstrate that, superior to photothermal excitation, this new configuration avoids direct heating of the microcantilever, thus minimizing undesired thermal effects on the vibration of microcantilever, while still keeps the advantage of being a remote, non-contact excitation method. We also measured the vibration amplitude of the microcantilever as a function of distance between the microcantilever and the Al foil and found that the amplitudes decay gradually according to the inverse distance law. This method is universal and can be adopted in bio-microelectromechanical systems (BioMEMs) for the detection of small signals where detrimental thermal effects must be avoided.

Microcantilever sensors have received considerable attentions for their ultrahigh sensitivity on the detection of small signals, such as mass^1^, forces^2^, chemicals^3^, and biological species^4^. A prerequisite for these applications lies on effective excitation of microcantilever to vibrate at the resonant frequencies with high quality factors (*Q*). Up to date, techniques most widely adopted for effective excitation of microcantilevers include piezoelectric actuation^5^, electrostatic actuation^6^, and magnetic actuation^7^. However, they are all near-field techniques, require usage of bulky components, or involve complex microfabrication processes, which have greatly hindered their applications. Recently, excitation methods by using acoustic waves^8^ and photothermal effects^9^ are particularly of interests due to advantages provided by remote, non-contact interactions. Both methods are considered as far field techniques with technological potentials for applications in harsh situations where other techniques are not easily accessible. However, conventional acoustic excitation requires usages of bulky piezoelectric or magnetic units to generate acoustic waves. Photothermal excitation involves undesired effects arisen from laser/microcantilever interactions. For example, laser will inevitably heat up the microcantilever, and samples vulnerable to light exposure or heat, e.g. biological species, could be damaged by laser^10^. Besides, neither transparent materials, e.g. glass or wide gap semiconductors, nor infrared light sources (*E* < 1.12 eV) can be adopted to make silicon based microcantilever sensors to take advantage of photothermal excitation. In this article, we show a new experimental configuration for efficient microcantilever excitation by taking advantages of both acoustic and photothermal excitations by using photoacoustic effect through an aluminum (Al) foil closely placed a microcantilever. This new method avoids direct heating of the microcantilever, thus minimizing undesired thermal effects on the vibration of microcantilever.

## Results and Discussion

**Figure 1:**
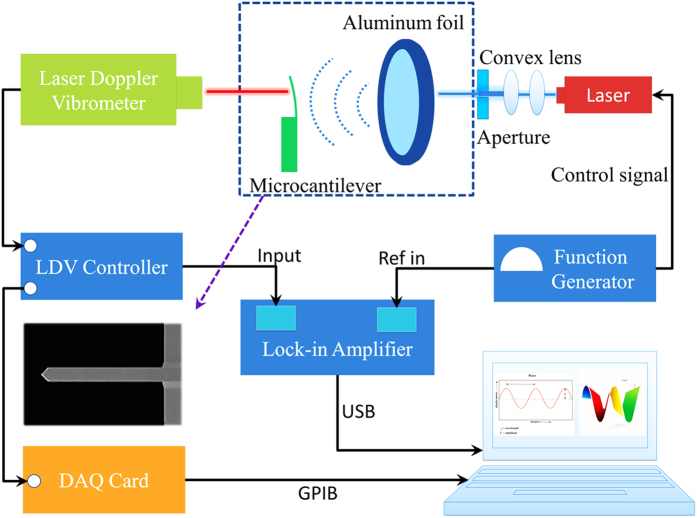
Diagram of the experimental setup for photoacoustic excitation.

Figure 1 shows the experimental setup used in this investigation. The microcantilever was actuated by acoustic waves from the vibrating Al foil induced by laser interaction. The radius of the Al foil was carefully adjusted such that the frequency response is flat in the resonant domain of the microcantilever. The vibration behaviors of the microcantilever were recorded and analyzed by studying the Doppler effect, the frequency change of the laser beam in response to a vibrating object. The apparatus is capable of resolving a displacement in the order of 1 pm in the frequency range of 2 Hz −2 MHz^11^.

### Method validation

**Figure 2:**
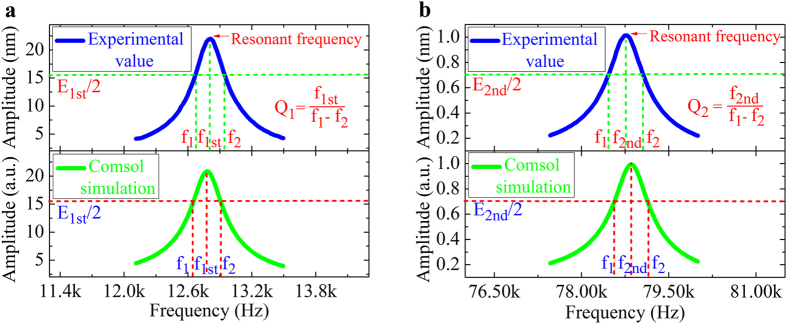
(a) Measured (upper panel) and simulated (lower panel) frequency response curves for the 1^st^ flexural resonant vibration of the microcantilever. **(b)** Measured (upper panel) and simulated (lower panel) frequency response curves for the 2^nd^ flexural resonant vibration of the microcantilever.

Figure 2 shows the measured and calculated frequency response curves of the first two flexural resonant frequencies of the microcantilever obtained by photoacoustic excitation. The measurement was carried out by sweeping the frequency with a step of 5 Hz. The 1st and 2nd flexural resonant frequencies were determined to be 12.80 kHz and 78.78 kHz, respectively. The measured values agree well with our theoretical calculations by using the classical beam theory^12^. The theoretical values for the 1st and 2nd flexural modes are found to be 13.11 kHz and 82.16 kHz, respectively, by taking the mode-dependent coefficients *k*_*1*_ = 1.875 and *k*_*2*_ = 4.694, and *ρ* = 2.329 g ∙ cm^−3^, *E* = 130 GPa for (100) silicon. The results agree well with the experimental values with a relative error less than 5%.

To further validate the method, the results were also compared with finite element method (FEM) simulation by using COMSOL Multiphysics, as shown in Fig. 2 (lower panels). The model consists of a 3D microcantilever fixed at one end, which is subjected to a boundary load to produce deflection. The microcantilever is defined as silicon and has the same geometries as that used in our experiments. The Rayleigh damping matrix, [*C*] = *α*_*R*_[*M*] + *β*_*R*_[*K*], was used to describe the damping behaviors of the system^13^,^14^, where *α*_*R*_ and *β*_*R*_ are coefficients associated with the mass (*M*) and stiffness (*K*) matrices, respectively. We first calculated the values of *α*_*R*_ and *β*_*R*_ by analyzing the damping behaviors of each resonant mode in air by using^15^


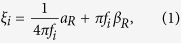


where *ξ*_*i*_ and *f*_*i*_are the damping ratios and resonant frequencies of mode *i,* respectively. *ξ*_*i*_ can be obtained from experiments and *f*_*i*_ can be calculated by FEM. The frequency response curves of the first two resonant modes simulated by FEM agree very well with the experimental data. Minor discrepancies could be attributed to unavoidable effects arose from uncertainties associated with 1) local temperature and humidity variations, 2) geometry determination of the microcantilever, 3) the goodness of the Rayleigh approximation. Taking all these limitations into account, the simulation results are quite satisfactory when compared to the experimental data.

#### Comparisons between photothermal and photoacoustic excitations.

**Figure 3:**
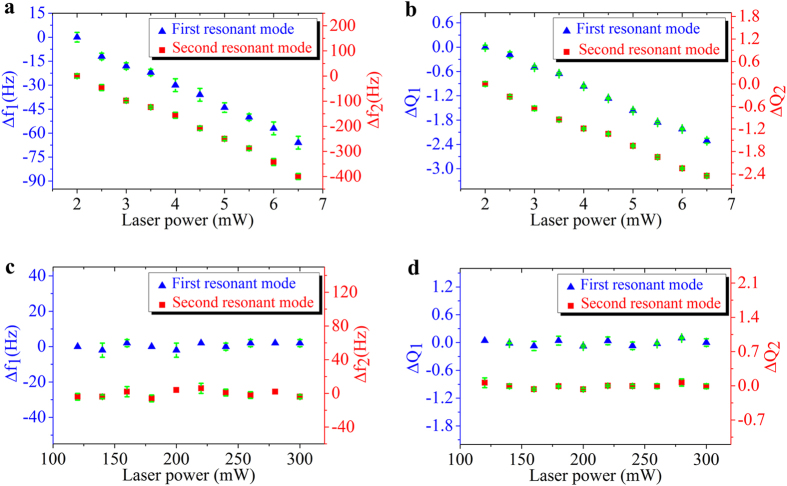
(a) The variation of the first two flexural resonant frequencies of the microcantilever obtained by photothermal excitation with the laser power increased from 2 mW to 6.5 mW gradually with a step of 0.5 mW. **(b)** The corresponding variation of the quality factors (*Q*) of the first two flexural resonant modes obtained by photothermal excitation. **(c)** The variation of the first two flexural resonant frequencies obtained by photoacoustic excitation with the laser power on the Al foil varied between 120 mW and 300 mW. **(d)** The corresponding variation of the quality factors of the first two flexural resonant modes obtained by photoacoustic excitation.

We investigated the effects of laser power on the photothermal excitation of a silicon microcantilever. Figure 3a shows the variation of the resonant frequencies of the microcantilever as a function of the laser power. Increasing the laser power from 2 mW to 6.5 mW resulted in a linear reduction of the resonant frequencies. We found a 66 Hz decrease for the 1st resonant frequency and a 400 Hz decrease for the 2nd resonant frequency, respectively. Figure 3b shows the dependence of quality factors of the microcantilever on the laser power. Increasing the laser power also results in reduced quality factors^16^. We found a reduction of 2.31 and 2.15 for the 1st and 2nd resonant modes, respectively. Reducing the laser power will lead to recovery of the resonant frequencies to their original values. As a result, we expect that the deflection of the microcantilever is on the elastic region. In comparison, the photoacoustic excitation shown in Fig. 3 will eliminate undesired photothermal effect. The vibration of the Al foil produces acoustic waves^17^ that excite the microcantilever to vibrate. As the microcantilever was isolated by the Al foil from the laser beam, optical interference and heat noise on the microcantilever vibration were eliminated. It can be seen from Fig. 3c,d that that the first two flexural resonant frequencies and quality factors were much more stable than direct photothermal excitation. Note that there are some experimental errors in the measured vibration amplitudes. As the laser Doppler vibrometer has a displacement resolving capacity of ~1 pm, we believe that most errors arise from uncertainties associated with environment fluctuations, e.g. background sound noise, temperature, humidity and distance determination.

### Shift of resonant frequencies

The shift in the resonant frequency can be attributed to the temperature-dependent variations of material properties, e.g. the coefficient of thermal expansion (*CTE*, *α*) and Young’s modules (*E*). The vibration of a one-end clamped rectangular microcantilever can be described by the Euler–Bernoulli beam theory, with the resonance frequencies given by^18^


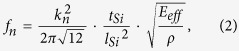


where *k*_*n*_, *ρ* and *E* are the mode-dependent coefficient, the density and the Young’s modules, respectively. For a microcantilever with a *CTE* of *α*, a thickness of *t*_*Si*_ and a length of *L*_*Si*_, a rise of temperature by Δ*T* will lead to an increase of *t*_*Si*_ and *L*_*Si*_ by (1 + *α*Δ*T*), a decrease of *ρ* by (1 +* α*Δ*T*)^3^. As a result, the resonant frequencies will increase by (1 + *α*Δ*T*)^1/2^. Note that the Young’s modulus, *E*, also depends on temperature with a relationship given by:


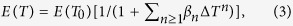


where β is the thermal coefficient of the elastic compliance constant (*S*). The 1st order β for silicon is (63.6 ± 0.6) × 10^−6^ K^−1^, while the higher order β are orders of magnitude smaller and thus can be ignored^15^. One then can obtain that, for a temperature variation of Δ*T*, the resulting resonant frequencies of a microcantilever will be proportional to 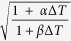. As the *CTE* of silicon is 2^6^ × 10^−6^ K^−1^ 20, much smaller than the 1st order β, photothermal excitations will reduce the resonant frequencies. This argument agrees with the experimental observations shownin Fig. 3a.

According to above discussion, we can also deduce that the ratio of the varied resonant frequencies (Δf_*2*_/Δf_*1*_) upon temperature change that is given by:


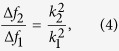


by substituting the mode-dependent coefficients *k*_*1*_ = 1.875 and *k*_*2*_ = 4.694 to Equation (4), we find a ratio of 6.267, which agrees well with the ratio (6.181) between the slopes of the two linear fitted lines shown in Fig. 3a. Obviously, the resonant frequencies of all higher order flexural modes will decrease even much faster than that of the 1st resonance mode.

### Change of quality factors

The energy loss of a vibrating microcantilever is dominated by attachment loss (*AL*), surface loss (*SL*), thermoelastic dissipation (*TD*) and air damping (*AD*). The total quality factor for the microcantilever can be expressed as:


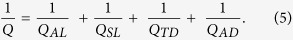


For the attachment loss, it is mainly caused by the microcantilever interaction with the support and independent of temperature [*Q*_*AL*_ = 0.34(*l*_*Si*_/*t*_*Si*_)^3^]^19^. Surface loss is mostly caused by surface stress, originated from surface absorbates^20^ or defects^21^, which is usually quite small in comparison with other loss mechanisms in atmosphere^22^. Thermoelastic dissipation involves energy loss induced by the oscillating transversal heat flow in the beam^23^. The energy loss depends on the materials, the geometry of the beam, and the working temperature, and the quality factor can be written as^24^:


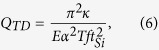


where *κ* is the thermal conductivity of silicon. Our calculations indicate that *Q*_*TD*_ (~10^7^) is orders of magnitude greater than the experimental values (<10^2^), and thus can be ignored.

The energy loss associated with air damping depends on the air pressure, which can be divided into three different regions: the intrinsic, the molecular, and the viscous region. In this present study, the air damping was in the viscous region. For a microcantilever with a quadratic cross section, a previous study^25^ showed that the quality factor can be approximated by an oscillating sphere with radius *R* with:


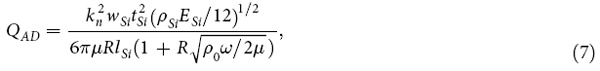


where *R*, *μ* and *ω* are the radius of the spheres, the dynamic viscosity of air and the angular frequency of the vibrating beam, respectively. A detailed analysis reveals that *Q*_*AD*_ will dominate the total quality factor (*Q*) and will decrease upon temperature rise, in agreement with the experimental results shown in Fig. 3b.

### Power dependence

**Figure 4:**
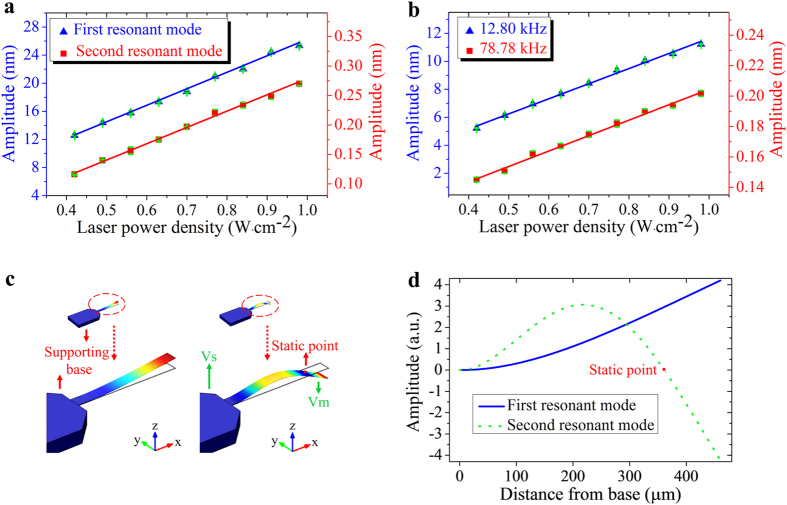
(a) Measured microcantilever deflection as a function of laser power for the first two resonant frequencies by photoacoustic excitation. **(b)** Measured deflection of the Al foil as a function of laser power at the first two resonant frequencies of the microcantilever. **(c)** FEM simulation of the first two flexural modes of the microcantilever. **(d)** Amplitude along the length of the fixed point of the microcantilever beam for the first two resonant modes.

Figure 4a shows the variation of the vibration amplitude of the microcantilever by photoacoustic excitation as a function of the laser powers. All the experiments shown here have been repeated several times. Figure 4a reveals that the vibration amplitudes of the microcantilever increases linearly from 12.58 nm to 25.35 nm and from 0.12 nm to 0.27 nm for the 1st and 2nd flexural modes, respectively, upon variation of laser power densities on the Al foil. A verification experiment also reveals the vibration amplitudes of the Al foil are linearly dependent on the laser power (Fig. 4b). However, the excitation mechanisms for the two resonant modes are different. As shown in Fig. 4c, the 1st resonant mode features deflection of the microcantilever in the same direction, while the 2nd resonant mode features a static point, around which the beam vibrates in opposite directions (Fig. 4d). Since the microcantilever is much smaller than the wavelength of the acoustic wave, the microcantilever can be approximately treated as a point object. As a result, the acoustic pressure radiated on the microcantilever features a uniform phase distribution along the microcantilever beam, so that only the 1st resonance can be effectively actuated while the 2nd resonance is not pronounced. However, it should be pointed out that the microcantilever is attached to the supporting base, which is also under the sound radiation. The vibration of the base can also drive the microcantilever to vibrate in the opposite direction and the 2nd resonant mode could still be actuated^26^. This is equivalent to exerting two forces with 180° phase difference on the left-hand side and right-hand side of the static point, respectively^27^.

### Distance dependence

**Figure 5:**
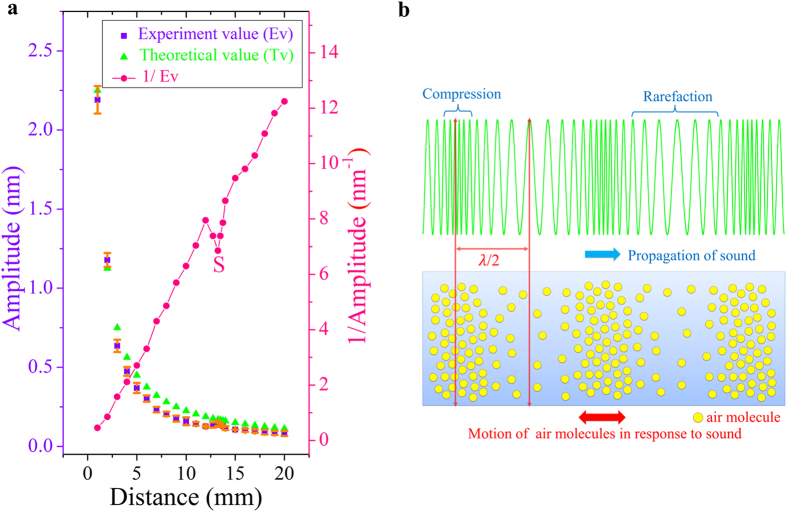
(a) Measured and calculated vibration amplitudes as a function of distance between the microcantilever and the Al foil. The laser power radiated on the Al foil was 200 mW. **(b)** The horizontal displacement of the air particles under the interaction of the standing wave. The motion of the air particles is in the direction parallel to the direction of energy transportation of the longitude wave.

We also measured the vibration amplitude of the microcantilever as a function of seperation between the microcantilever and the Al foil. The blue squares in Fig. 5a show the vibration amplitude of the 1st flexural mode as a function of the seperation between the microcantilever and the Al foil. We find that the vibration amplitude falls according to the inverse distance law, as shown by the green triangles in Fig 5a. The deviation could be related to the small size of the microcantilever. In the experiments, we used a microcantilever that is 10 times stiffer than that in Fig. 2a, such that the vibration amplitudes are smaller.

Note that there is a significant increase of displacement at 1.325 cm, exactly one-half wavelength of the sound wave. It becomes more pronounced by taking the reciprocal values for the experimental data, as shown by Point S in Fig. 5a. This is because the interference of the incident and sound waves reflected by the microcantilever. From a microscopic point of view, the vibration of the Al foil produces periodic distribution of air pressure between the microcantilever and the Al foil, as shown in Fig. 5b. When the separation distance between the microcantilever and the Al foil is equal to one-half of the sound wavelength, the energy can be more easily transferred to the microcantilever, resulting in increased vibration amplitude. Besides, a careful designed waveguide or acoustic lens could also be adopted to concentrate the sound wave onto microcantilever to increase the vibration amplitude.

## Conclusions

In summary, we develop a new photoacoustic method for efficient microcantilever excitation. The method is a promising alternative to conventional methods as a remote excitation method for microcantilever based sensors. Compared with traditional photothermal excitation, our results indicate that this new method can effectively eliminate the influence of thermal effect. We also investigate the relationship of the displacement of the microcantilever with 1) the laser power and 2) the distance between the microcantilever and Al foil. We believe that this new method is general and have significant technical implications for the detection of small signals in systems that are sensitive to heat, e.g. cells or most biological systems.

## Methods

The main structure shown in Fig. 1 consisted of a microcantilever (SICON, length *l*_*Si*_ = 460 μm, width *w*_*Si*_ = 49 μm, thickness *t*_*Si*_ = 2.3 μm) and an aluminum (Al) foil (thickness *t*_*Al*_ = 50 μm), as shown in the dashed box of Fig. 1. The Al foil was circular in shape, mounted on a perforated black Al plate with diameters ranging from 3 mm to 10 mm. It was coated with a layer of graphite to increase laser absorption. The Al foil was excited to vibrate by photothermal forces generated by a modulated laser beam controlled by an arbitrary waveform generator (Model 33220a, Agilent, USA). The laser beam (single mode Gaussian beam) was expanded and shaped into a beam with uniform irradiance by two convex lens. We then use a circular aperture to adjust the diameter of the laser spot to completely cover the Al foil. The power density of the laser beam was calibrated by an optical power meter (model PD300-UV-193 ROHS, OPHIR, Israel). The distance between the Al foil and microcantilever is maintained to be 1 mm. The vibration of the microcantilever was monitored by a laser Doppler vibrometer (Model OFV-5000/534, Polytec, Germany), equipped with a lock-in amplifier (Model EG&G 7260, Signal recovery, USA). The experimental data were collected by a data acquisition card (Model PCI-6111, NI, USA) and processed by a PC.

## Additional Information

**How to cite this article**: Gao, N. *et al*. Microcantilever Actuation by Laser Induced Photoacoustic Waves. *Sci. Rep.* **6**, 19935; doi: 10.1038/srep19935 (2016).
